# Erectogenic Effects of *Clerodendron capitatum*: Involvement of Phosphodiesterase Type-5 Inhibition

**DOI:** 10.1155/2012/137386

**Published:** 2011-06-13

**Authors:** Siddig Ibrahim Abdelwahab, Abdelwahab Hassan Mohamed, Osama Yousif Mohamed, Mahjoub Oall, Manal Mohamed Elhassan Taha, Syam Mohan, Mohamed Ibrahim Noordin, Mohd Rais Mustafa, Khalid M. Alkharfy

**Affiliations:** ^1^Faculty of Medicine, University of Malaya, Petaling Jaya, Kuala Lumpur 50603, Malaysia; ^2^Medicinal and Aromatic Plants Institute, National Centre for Research, Khartoum 1111, Sudan; ^3^Department of Pharmacology, Faculty of Pharmacy, University of Khartoum, Khartoum 1111, Sudan; ^4^UPM-MAKNA Cancer Research Laboratory, Institute of Biosciences, University of Putra Malaysia, Serdang, Selangor 43400, Malaysia; ^5^Department of Clinical Pharmacy, College of Pharmacy, King Saud University, Riyadh 11451, Saudi Arabia

## Abstract

*Clerodendron capitatum* (Willd) (family: verbenaceae) is locally named as Gung and used traditionally to treat erectile dysfunction. Therefore, the current study was designed to investigate the erectogenic properties of *C. capitatum*. The relaxation effect of this plant was tested on phenylephrine precontracted rabbit corpus cavernosum smooth muscle (CCSM). The effects of *C. capitatum* were also examined on isolated Guinea pig atria alone, in the presence of calcium chloride (Ca^2+^ channel blocker), atropine (cholinergic blocker), and glibenclamide (ATP-sensitive K^+^ channel blocker). These effects were confirmed on isolated rabbit aortic strips. The extract, when tested colorimetrically for its inhibitory activities on phosphordiesterase-5 (PDE-5) *in vitro* towards *p*-nitrophenyl phenyl phosphate (PNPPP), was observed to induce significant dose-dependent inhibition of PDE-5, with an ID_50_ of 0.161 mg/ml (*P* < .05). In conclusion, our results suggest that *C. capitatum* possesses a relaxant effect on CCSM, which is attributable to the inhibition of PDE-5, but not mediated by the release calcium, activation of adrenergic or cholinergic receptors, or the activation of potassium channels.

## 1. Introduction

Erectile dysfunction is a serious clinical problem in adult men. The malfunction of penile erection could be due to impaired relaxation of the smooth muscle related to the increase in blood flow into the spaces of the *corpus cavernosum *[1]. Inhibition of cellular enzyme, phosphodiesterase-5 (PDE-5), reduces cyclic guanylate monophosphate breakdown, promoting vascular relaxation in the corpora cavernosa and penile erection during sexual stimulation [2]. Since several synthetic drugs commonly used in erectile dysfunction are associated with undesirable side effects, there is rising interest in discovering new effective drugs. Various reports on natural products with vasodilating effects on corpus cavernosum smooth muscle appeared recently [3, 4]. 

*Clerodendron capitatum *(Willd) (family: verbenaceae), locally named as Gung in Sudan, is an indigenous tropical African plant, which grows fast, erect, well branched and grows up to 0.5–2 m high [5]. In Sudan, the roots of this plant are used traditionally in the management of male erectile dysfunction [6, 7]. In Nigeria, this plant is used to treat diabetes mellitus, obesity, and hypertension [5]. The genus *Clerodendron *is reported to demonstrate versatile biological activities such as antitumorgenic [8, 9], hypoglycemic, hypolipidemic [10], hepatoprotective activity against CCl_4_-induced liver injury in rats [11, 12], anti-inflammatory [13–15], radical-scavenging activity [12, 16–18], antidiarrhoeal [19], antinociceptive, and antipyretic effects [14]. 

No phytochemical investigation was conducted on *C. capitatum *to isolate pure compounds. Nevertheless, the presence of saponins, alkaloids, flavonoids, glycosides, and reducing sugars was confirmed by simple qualitative methods [5]. To the best of our knowledge, there is only one paper published regarding the phytomedicinal properties of *C. capitatum* by Adeneye et al. [5]. Therefore, an extensive pharmacological investigation is needed to explore the biological activities of this plant associated with its folk phytotherapy for erectile dysfunction. Consequently, the current study was designed to investigate the erectogenic properties of *C. capitatum*.

## 2. Material and Methods

### 2.1. Plant Material

The plant *C. capitatum* was collected by a team from Medicinal and Aromatic Plants Research Institute (MAPRI), National Center for Research (NCR), Sudan during their scientific trip to Nuba Mountains, Kordofan State, Sudan. The plant was identified and authenticated by Dr. G. E. B. Elgazali, Department of Chemistry, Production and Classification, MAPRI, NCR. A voucher specimen was prepared and deposited in the Herbarium of MAPRI.

### 2.2. Extraction of Plant Material

The roots of the plant were coarsely air-dried in the shade and kept in clean plastic container for the work. The powdered roots (300 gm) of the plant were exhaustively extracted with methanol using Soxhlet apparatus. The extract was then evaporated under reduced pressure and kept in a refrigerator for further biological investigations. The percentage yield of the powdered roots was 9.67%. 

#### 2.2.1. Sample Preparation

Physiological salt solution was used as a vehicle to dissolve the *C. capitatum* root extract freshly prior to addition into the tissue bath, with a concentration of 10 mg/mL. Vehicle when used as control did not produce any contracting or relaxant effects into all tissues preparations.

### 2.3. Animals

Animals were obtained from the Experimental Animal House (EAH), MAPRI, NCR, Sudan. The animals were given standard animal feeding and tap water *ad libitum*. This study was approved by the Ethics Committee for Animal Experimentation, EAH, MAPRI, NCR.

### 2.4. Rabbit Corpus Cavernosum Smooth Muscle Preparation

Sexually mature male New Zealand White rabbits (4 ± 0.5 Kg) were used. This experiment was carried out following the method described by Chuang et al. [24]. The animal was sacrificed, and penectomies were performed to isolate the corpus cavernosum smooth muscle (CCSM). CCSM strips were isolated from the enveloping *Tunia albuginea* in cool physiological salt solution (PPS). A thread was attached at each end of the muscle strip, which was mounted in a 25 mL water-jacketed tissue bath containing PSS at 37°C and bubbled continuously with air. The tissues were equilibrated for at least 45 minutes and then stretched incrementally until tissue strips produced the optimal length, which occurred at forces of 2-3 grams in the unstimulated tissues. CCSM strips then contracted with phenylephrine (250 *μ*M). After a peak force was achieved, different concentrations of *C. capitatum* were added to tissue bath. The responses were recorded with isometric transducer. Results were expressed as percentage of phenylephrine-contracted CCSM. Findings of *C. capitatum* were compared to sildenafil, a type-5 CGMP phosphodiesterase inhibitor.

### 2.5. Guinea Pig Atrium Preparation

Guinea pigs of either sex (300–500 g) were used in this study. The pericardium was carefully removed. The atria were separated from the ventricles carefully by a cutting at the atrioventricular septum. A thread was attached to the tip of each atrium and the preparation was mounted on a tissue holder and transferred to 25 mL organ bath filled with aerated Ringer-lock's solution maintained at 30°C. The upper thread was attached to isometric transducer. To ensure steady preparation three readings of the spontaneous atrial rate were made before extract administration. To investigate the mechanism *C. capitatum* on atrium atropine, calcium chloride, and glibenclamide were used.

### 2.6. Rabbit Aortic Strip

A rabbit of local strain (1.75 ± 0.25 kg) was used. The rabbit was killed by neck dislocation and exsanguinated. The chest was opened, the internal viscera were pulled aside, and the aorta was exposed. The tissue was transferred to a petri dish containing aerated Kreb's solution. The aorta was located over a large plastic canulla, the surrounding fat and connective tissues were removed, and aorta was cut spirally to produce continuous strip. A thread was tied to each end of the strip (3-4 cm), and one end was fixed to tissue holder. The preparation was transferred to 25 mL organ bath filled with aerated Kreb's solution maintained at 37°C. The isometric contractions were recorded using isometric transducer connected to oscillographic recorder. The preparation was allowed to equilibrate under 1.5–2 gm tension for at least 45 minutes. Before adding the drugs, adrenaline and plant extract were left in tissue for two minutes.

### 2.7. Effect of *C. capitatum* on Phosphodiesterase Type-5

This experiment was carried out following the method described by Kelly and Butler [20]. Two mg of the enzyme PDE-5 (Sigma Aldrih, USA) were dissolved into 20 mL Trizma buffer (pH 8.7) maintained at 37°C. From the substrate, *p*-nitrophenyl phenylephosphate (Sigma Aldrih, USA), 55.84 mg were dissolved in 20 mL of Trizma buffer to produce 5 mM working solution. The substrate, enzyme, and plant methanolic extract solution were immersed separately in a water bath at 37°C for 10 min to equilibrate. The extract (0.5 mL) was mixed with the enzyme solution (1 mL) and incubated in a water bath maintained at 37°C to allow the extract to occupy the active site of the enzyme. Mixture was then transferred to a clean cuvet, and using spectrophotometer (Jenway 6305 UV/VIS spectrophotometer) the absorbance of the mixture (*V*°) at 400 nm was red. This procedure was repeated using different concentrations of the extract. The mixture (Enzyme with different concentration of the extract) was added to the substrate solution (5 mM) left to stand for 30 sec, and absorbance of each concentration was determined (*V* ^1^) at the same wavelength.

### 2.8. Statistical Analysis

All experimental data was expressed as mean ± SD. Ordinary least squares regression were performed to calculate the ID_50_ and dose dependency. Level of significance was set as *P* < .05.

## 3. Results

### 3.1. Relaxant Effects of *C. capitatum* Extract on CCSM

**Figure 1:**
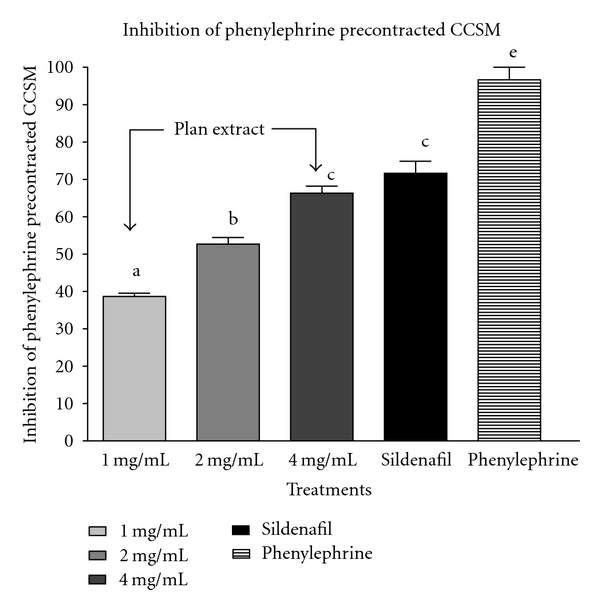
Relaxant effect of the methanol extract of C*. capitatum *on phenylephrine (250 *μ*M) precontracted *corpus cavernosum *strips. *Means with different alphabets are significantly different from the control.

This study investigated the relaxant effects of *C. capitatum* extract and sildenafil on corpus cavernosum smooth muscle (CCSM) preparation, to clarify mechanistically the effect of this plant in the penile erection. As demonstrated in Figure 1, addition of *C. capitatum* extract caused a significant (*P* < .05) concentration-dependent relaxation of CCSM that had been precontracted with phenylephrine. The maximal relaxation and the *C. capitatum* concentration required to produce 50% relaxation being 67 ± 8% and 4.0 mg/gut bath (25-mL), respectively. Treatment of tissues with sildenafil (2 mg/gut bath; 25-mL) decreased the contractive responses to phenylephrine, with the maximal relaxation being only 76 ± 12.5%.

### 3.2. Influence of *C. capitatum* Extract on Cardiac Muscle

**Figure 2:**
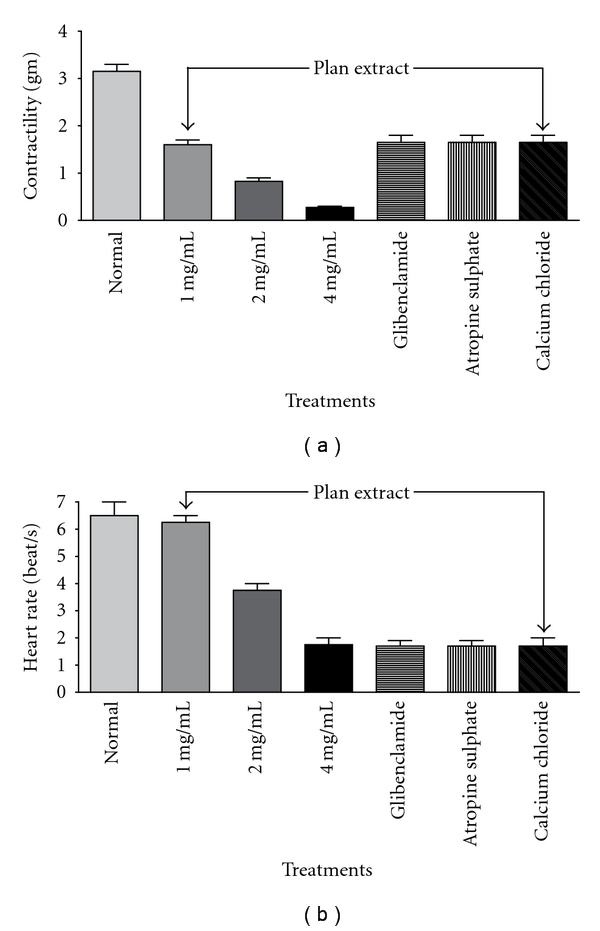
Concentration-dependent relaxation of Guinea pig atria induced by *C. capitatum*. Administration of the extract in a dose of 1, 2, and 4 mg/mL (gut bath) depressed the contractility (a) and the rate (b) of the atria. Administration of glibenclamide (500 *μ*g/25-mL), CaCl_2_ (450 mM/25-mL), and atropine (5 *μ*g/25-mL) did not reverse the depression.

*C. capitatum* extract when added to atrial preparation in doses of 1, 2, and 4 mg/25-mL (gut bath) depressed clearly (*P* < .05) the contractility and the rate of isolated atrium (Figure 2, *n* = 6). In about 15 min, the inhibitory effect of the extract achieved its maximum. To investigate that cardiac inhibitory effect, preincubation of a cholinergic blocker [atropine sulphate, (5 *μ*g/25-mL, gut bath)] failed to inhibit the relaxant effect of *C. capitatum*. It is also observed that simultaneous administration of CaCl_2_ (450 mM) did not reverse the plant inhibitory effect. Moreover, prior addition of glibenclamide in a dose of 500 *μ*g/25-mL failed to overturn the effect of the plant extract. This study showed that the plant extract was able to induce nonadrenergic-noncholinergic effects in penile corpus cavernosum. As cardiac muscles are known to specific sites for PDE type 5, it could be suggested that the pharmacological effects of *C. capitatum* is due to the inhibition of cGMP hydrolysis.

### 3.3. Effect of *C. capitatum* Extract on Rabbit Aortic Strip

To further investigate the nonadrenergic-noncholinergic relaxant effect of *C. capitatum*, rabbit aortic strip was used. *C. capitatum*, at doses of 1, 2, and 4 mg/25-mL (gut bath), did not produce any pharmacological activities on rabbit aortic strip (*n* = 6). Adrenaline achieved the maximal contracture in two-minute time when added in a dose of 2 mg/mL, gut bath. In the presence of *C. capitatum* extract at doses of 1, 2, and 4 mg/25-mL (gut bath), adrenaline was not blocked. These results further suggest that the plant extract did not interfere with activating or blocking the adrenergic receptors.

### 3.4. Determination of PDE-5 Activity

Table 1 shows the results of different concentrations of *C. capitatum* on the *in vitro* enzymatic hydrolysis of PNPPP using PDE-5. Using ordinary least squares regression, double reciprocal plot was performed for the enzyme inhibition (1/Extract concentration versus 1/Absorbance) to calculate the ID_50_ and dose dependency. The enzymatic hydrolysis of PNPPP, when recorded spectrophotometrically at 400 nm measuring the appearance of *p*-nitrophenol (yellowish color), revealed that the reaction was dose dependent. It is observed that the plant extract when added to the enzyme (PDE-5) markedly (*P* < .05) and dose dependently (*r* = − 0.983, *P* < .05) decreased the rate of enzymatic hydrolysis of PNPPP. The calculation revealed that ID_50_ of *C. capitatum* is 0.161 ± 0.08 mg/mL (gut bath) as shown in Table 1.

## 4. Discussion

In the present study, we have investigated the effect of the methanolic extract of the roots of *Clerodendron capitatum* on the relaxation of phenylephrine precontracted rabbit corpus cavernosum smooth muscle as well as on PDE-5 hydrolytic activity. These inhibitory activities of *C. capitatum* in penile corpus cavernosum were also confirmed using isolated animal tissues such as Guinea pig atria and rabbit aorta. This research was conducted based on the popular folk use of *C. capitatum* as aphrodisiac and neurotonic tonic in Sudanese traditional medicine.

Cyclic guanosine 3′,5′-monophosphate (cGMP) is an important second messenger within cells. At the cellular level, cGMP is synthesized by guanylyl cyclase and degraded by phosphodiesterases (PDEs). Roles of cGMP and PDEs are related to many signalling pathways. Therefore, many physiological functions, that is, cardiac contractility and smooth muscle relaxation are controlled by PDEs. PDEs have been classified to 11 families by different specificities and sensitivities to endogenous and exogenous substances [21–23]. Penile Erection is a hemodynamic event regulated by smooth muscle relaxation of CCSM after elevation of cellular cGMP. Elevation of cGMP levels by PDE-5 inhibitors is rationale for the therapeutic approach for inducing penile erection in individuals with erectile dysfunction [24].

*C. capitatum* significantly potentiated the relaxation of rabbit cavernosal strips. The PDE-5 inhibitor sildenafil also prolongs the relaxations of cavernosal tissue and bovine penile arteries. Furthermore, phenylephrine leads to concentration-dependent relaxations in animal and human corpus cavernosum that is enhanced by sildenafil [25]. Therefore, our data showing that *C. capitatum* also caused marked in the response curves to phenylephrine in the rabbit corpus cavernosum indicate that these actions are due to inhibition of PDE5 in the cavernosal tissue [25, 26]. Our data showed also the *in vitro* enzymatic hydrolysis of *p*-nitrophenyl phenylephosphate (PNPPP) by PDE-5 was inhibited significantly by *C. capitatum*. The hydrolytic activity was assayed by monitoring the formation of *p*-nitrophenol following enzymatic hydrolysis of PNPPP [20, 27, 28]. This experimental approach has been applied elsewhere for detection of biological activities of some plant extracts towards PNPPP [29]. The pattern of relaxation produced by the extract that mimics the effect of sildenafil coupled with enzyme substrate inhibitory effect of the extract suggested that phosphodiesterase inhibition was the possible mode of action of the extract on CCSM. These findings support the hypothesis that the ultimate accumulation of cGMP due to inhibition of PDE-5 is the possible cause of relaxation of CCSM [25]. 

The inhibition of PDE-5 by *C. capitatum* pushed us to carry out *in situ* experiments on Guinea pig atria and rabbit aortic strips to verify the mechanism of *C. capitatum*. Our results on Guinea pig atria preparation showed that the relaxant effects of the plant extract were not interfered with cellular calcium ion mobilization since simultaneous administration of calcium chloride did not reverse its inhibitory effect on the myocardial muscle, however, the nature of the mechanism governing cross-bridge interactions with thin filaments and the resulting contraction in smooth muscle is depended on the Ca^+2^. There is consensus that activation by neurotransmitter or depolarization involves myosin light chain phosphorylation by the Ca^+2^ calmodulin-dependent kinase, enabling cross-bridge and cycling [24]. The effect of *C. capitatum* on Guinea pig atria might not be due to the competitive antagonism for the muscarinic acetylcholine receptors [30] since its action was not reverse by pre-incubation of atropine sulphate. Moreover, prior addition of glibenclamide, an ATP-sensitive K^+^ (K^+^ ATP) channel blocker, failed to reverse the effect of plant. Our above-mentioned results also showed that the roots methanolic extract relaxant properties on CCSM were not due to adrenergic blockage, since it did not antagonize the stimulant effect of adrenaline on isolated rabbit aortic strips. 

From the current study, the plant extract showed high PDEs inhibitory effect in comparison to the standard PDE inhibitor, sildenafil. The plant also showed potent relaxation in precontracted rabbit cavernous strips. This pharmacological effect is suggested to be through the inhibition of PDE-5 since the effects of the extract did not interfere with atropine, calcium chloride, and glibenclamide. It could also be interesting to further isolate and elucidate the structures of PDEs inhibitor in these extracts.

## Figures and Tables

**Table 1:** The effect of *C. capitatum *extract on phosphordiesterase-5 *in vitro* towards *p*-nitrophenyl phenyl phosphate.

Treatment	% of relaxation*
*C. capitatum *0.30 mg/mL	75.1^a^ ± 2.7
*C. capitatum *0.15 mg/mL	43^b^ ± 1.9
*C. capitatum *0.075 mg/mL	34^c^ ± 2.5
*C. capitatum *0.04 mg/mL	31^d^ ± 4.9
*C. capitatum *0.02 mg/mL	15^e^ ± 3.7
Sildenafil 0.10 mg/mL	79^a^ ± 13.2

The controls with vehicle did not show any significant effect. Using ordinary least squares regression, double reciprocal plot was performed for the enzyme inhibition (1/Extract concentration versus 1/Absorbance) to calculate the ID_50_ and dose dependency.
